# Mapping a major QTL responsible for dwarf architecture in *Brassica napus* using a single-nucleotide polymorphism marker approach

**DOI:** 10.1186/s12870-016-0865-6

**Published:** 2016-08-18

**Authors:** Yankun Wang, Wenjing Chen, Pu Chu, Shubei Wan, Mao Yang, Mingming Wang, Rongzhan Guan

**Affiliations:** 1 State Key Laboratory of Crop Genetics and Germplasm Enhancement, Nanjing Agricultural University, Nanjing, 210095 China; 2 Jiangsu Collaborative Innovation Center for Modern Crop Production, Nanjing, Jiangsu China

**Keywords:** *Brassica napus*, Dwarf architecture with down-curved leaf mutant, Single-nucleotide polymorphism, Gene mapping

## Abstract

**Background:**

Key genes related to plant type traits have played very important roles in the “green revolution” by increasing lodging resistance and elevating the harvest indices of crop cultivars. Although there have been numerous achievements in the development of dwarfism and plant type in *Brassica napus* breeding, exploring new materials conferring oilseed rape with efficient plant types that provide higher yields is still of significance in breeding, as well as in elucidating the mechanisms underlying plant development. Here, we report a new dwarf architecture with down-curved leaf mutant (*Bndwf/dcl1*) isolated from an ethyl methanesulphonate (EMS)-mutagenized *B. napus* line, together with its inheritance and gene mapping, and pleiotropic effects of the mapped locus on plant-type traits.

**Results:**

We constructed a high-density single-nucleotide polymorphism (SNP) map using a backcross population derived from the *Bndwf/dcl1* mutant and the canola cultivar ‘zhongshuang11’ (‘ZS11’) and mapped the dwarf architecture with the down-curved leaf dominant locus, *BnDWF/DCL1*, in a 6.58-cM interval between SNP marker bins M46180 and M49962 on the linkage group (LG) C05 of *B. napus.* Further mapping with other materials derived from *Bndwf/dcl1* narrowed the interval harbouring *BnDWF/DCL1* to 175 kb in length and this interval contained 16 annotated genes. Quantitative trait locus (QTL) mappings with the backcross population for plant type traits, including plant height, branching height, main raceme length and average branching interval, indicated that the mapped QTLs for plant type traits were located at the same position as the *BnDWF/DCL1* locus.

**Conclusions:**

This study suggests that the *BnDWF/DCL1* locus is a major pleiotropic locus/QTL in *B. napus,* which may reduce plant height, alter plant type traits and change leaf shape, and thus may lead to compact plant architecture. Accordingly, this locus may have substantial breeding potential for increasing planting density.

**Electronic supplementary material:**

The online version of this article (doi:10.1186/s12870-016-0865-6) contains supplementary material, which is available to authorized users.

## Background

Traits related to plant height or compact plant type are very important due to their role in enhancing lodging resistance or the planting density in crops [1–4]. Certain key genes related to plant type traits have played very important roles in crop genetic improvement. In wheat, the *Rht* (Reduced height) genes controlling a key step in the signal transduction pathway of the growth hormone gibberellic acid (GA), have been utilized worldwide, bringing about the “green revolution” in crop production [1]. In rice, a semi-dwarf gene *sd1* that regulates a key step in the biosynthesis of GA [5–7] has proved extremely important in elevating harvest index and lodging resistance in worldwide rice production. However, the mechanism underlying the development of plant type or dwarfism is complex because many loci related to plant hormone biosynthesis and signal transduction [e.g., GA, brassinosteroid (BR) and auxin] and transcription factors, might determine plant height and architecture [8–15].

Rapeseed is one of the crops prone to lodging, which can lead to yield loss and difficulty in harvesting, and thus scientists have paid considerable attention to dwarfism in *B. napus* [16–19]. The dwarf gene *Bzh*, which was derived from the cultivar ‘Primor’ through chemical mutagenesis, was first identified and later mapped in 1995 [16, 17]. The dwarf gene *Bzh* has an additive effect, which may bring about a greater than 30 % reduction in plant height [16, 17]. Using this gene, several dwarf or semi-dwarf rapeseed cultivars, such as ‘Bienvenu-*bzh*’, ‘2405-*bzh*’ and ‘Darmor-*bzh*’ (with formally released genome sequences), have been raised. Muangprom et al. [20–22] identified and studied the dwarf gene *Brrga1-d* on the A06 chromosome of *Brassica rapa*, which encodes a DELLA protein, and transferred this to *B. napus* cultivars. Experiments have demonstrated that this gene may reduce plant height and elevate lodging resistance due to an altered GA signaling pathway. Later, the semi-dwarf gene *DS-1* in *B. napus* was mapped to chromosome A06. Molecular experiments have demonstrated that *DS-1* encodes a DELLA protein in which a single proline (P) to leucine (L) substitution in the VHYNP motif leads to dwarf mutation, a gain-of-function mutation in GA signaling [18]. In another study, the dwarfism of *B. napus banC.dwf*, was found to be controlled by one recessive gene that leads to insensitivity to exogenous GA3 [19]. In addition to the aforementioned dwarfism related to GA biosynthesis or signaling, dwarfism related BR signaling or other pathways has also been identified. The phenotype of the *B. napus* dwarf mutant ‘NDF-1’ was found to be controlled by a major gene possessing a mainly additive effect and a non-significant dominance effect, and a three-base mutation in the pyrimidine box (P-box) of the *BnGID1*promoter was found to be linked to its dwarf phenotype [23, 24]. Recently, the dominant *BnDWF1* locus on chromosome BnA09 has been found to be associated with the *B. napus* dwarf mutant, *Bndwf1* [25].

Although there have been numerous achievements in the exploration of dwarfism and plant type breeding in *B. napus*, exploring new materials that confer oilseed rape with efficient plant types leading to considerably higher yields is still of significance in breeding, as well as in elucidating the mechanisms underlying plant development. New materials with specific plant type traits, such as compact plant architecture and shorter plant height, are worthy of investigation. The present study describes a dominant dwarf architecture with down-curved leaf mutant (*Bndwf/dcl1*) isolated from an EMS-mutagenized *B. napus* line, together with its inheritance, gene mapping and effects on the agronomic traits. Additionally, to map the dominant *BnDWF/DCL1* locus and QTLs for plant type-related traits, we constructed a saturated SNP linkage map. Our findings may offer insight into elucidating the molecular mechanism underlying the dominant compact plant type and down-curved leaf phenotype and identification of the key gene controlling the plant height and down-curved leaf trait in *B. napus*.

## Results

### Performance of the dwarf down-curved leaf mutant

**Fig. 1:**
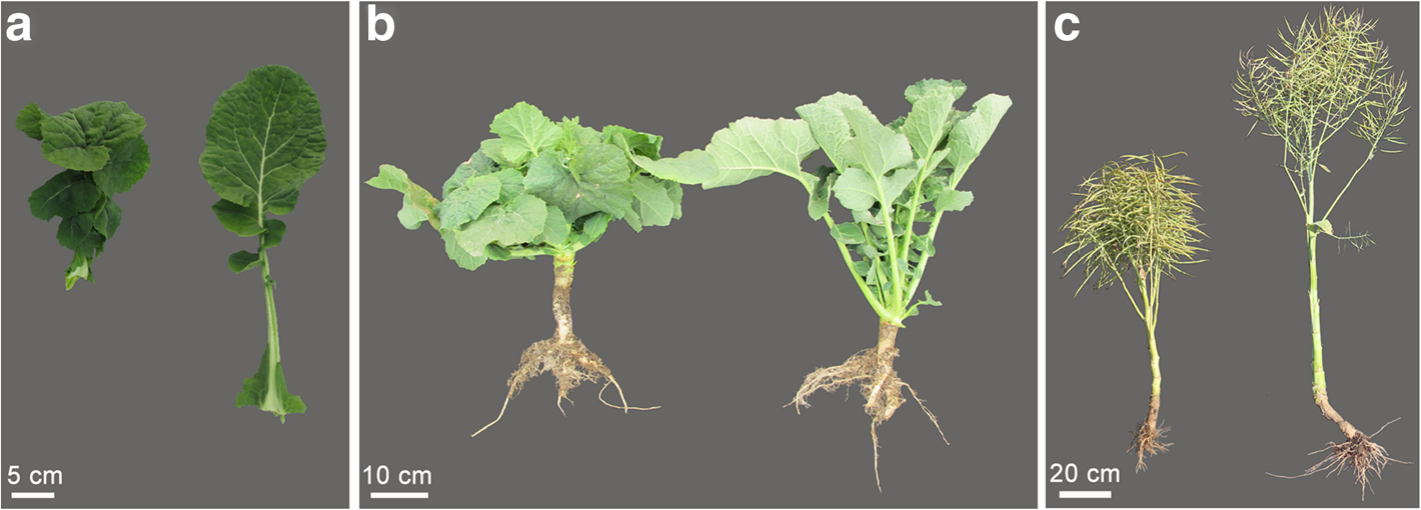
Morphological charactersof the *Bndwf/dcl1* mutant and its wild-type. **a** Leaf phenotype at the seedling stage of the *Bndwf/dcl1* mutant (*left*) and its wild-type (*right*). **b** Phenotype at the seedling stage of the *Bndwf/dcl1* mutant (*left*) and its wild-type (*right*). **c** Phenotype at the mature stage of the *Bndwf/dcl1* mutant (*left*) and its wild-type (*right*)

At the seedling stage, leaves of the *Bndwf/dcl1* mutant have a sharply down-curved and crinkled phenotype, with short petioles, which contrasts with the wild-type leaves that are normal with long petioles (Fig. 1a and b). The leaves of the adult *Bndwf/dcl1* mutant become slightly down-curved and crinkled before flowering, and are slightly down-curved but not crinkled after flowering. At the mature stage, the *Bndwf/dcl1* mutant plant exhibits a compact dwarf plant type, while the wild-type (Fig. 1c) exhibits a tall plant type. Plant height of the *Bndwf/dcl1* mutant was only 40–70 cm, which is considerably shorter than that of the wild-type (approximately 1.5 m).

### Inheritance of the dwarf architecture with down-curved leaf trait

The F_1_ plants of ‘ZS11’ × *Bndwf/dcl1* had down-curved leaves and dwarf plant height, indicating that the dwarfism and down-curved leaves are dominant traits. The plants in the BC_1_ population could be divided into two different groups: dwarf plants with down-curved leaves, and tall plants with normal flat leaves (Fig. 1). The 423 BC_1_ plants contained 223 dwarf plants with down-curved leaves and 200 normal plants with normal leaves. A chi-squared test indicated that the segregation pattern agreed with the Mendelian segregation ratio of 1:1 (Table 1). In the F_2_ population, the segregation obeyed the Mendelian segregation ratio of 1:3 (normal plants with normal flat leaves: dwarf plants with down-curved leaves) (Table 1). The reciprocal F_2_ population (RF_2_) was verified to have the same inheritance segregation mode as the F_2_ population (Table 1). Thus, we infer that the dwarf architecture with down-curved leaf trait is controlled by a single dominant gene.

**Table 1:** Inheritance of the dwarf architecture with down-curved leaf trait of *Bndwf/dcl1*

Population	No. of normal plants with normal leaves	No. of dwarf plants with down-curved leaves	Expected segregation	*χ* ^2^	*p* value
BC_1_	200	223	1 : 1	1.14	0.29
F_2_	126	319	1 : 3	2.43	0.12
RF_2_	97	247	1 : 3	1.71	0.19

Several plant type-related traits were investigated in the backcross population. It was found that the plants with down-curved leaves were consistently dwarf and compact in plant type. For plants with down-curved leaves, measurements for plant height, branching height, main raceme length and average branching interval, were all significantly smaller than those of the plants with normal leaves (Table 2). The plant height was on average reduced by approximately 60 % compared with the normal plants. Plant height was significantly correlated with branching height, main raceme length and average branching interval, with correlation coefficients *r* = 0.868**, 0.945** and 0.736** respectively (** denotes significant at the 0.01 level, see Additional file 1). These results clearly indicate that the down-curved leaf trait is accompanied by compact plant type architecture by reduced branching height, main raceme length and branching interval. However, the compact type of plant architecture does not necessarily indicate reduction in the number of the primary branches (Table 2). Additionally, in the BC_1_ population, the 1000-seed weight and yield per plant of dwarf plants with down-curved leaves were both significantly less than those of the normal plants with normal leaves (Table 2).

**Table 2:** Agronomic performance of the *Brassica napus* ‘ZS11’ × (‘ZS11’ × *Bndwf/dcl1*) BC_1_ population

Trait	‘ZS11’	*Bndwf/dcl1*	F_1_	BC_1_ population		
				Normal plants with normal leaves	dwarf plants with down-curved leaves	*p* value
Plant height	143.5 ± 7.0	49.3 ± 8.7	56.2 ± 2.6	122.5 ± 13.0	48.2 ± 8.0^b^	1.7E-237
Branching height	29.2 ± 3.5	9.9 ± 3.8	11.9 ± 3.8	34.0 ± 7.6	10.2 ± 3.3^b^	2.2E-155
Main raceme length	72.3 ± 6.6	24.4 ± 4.2	29.6 ± 5.1	57.9 ± 9.3	25.3 ± 6.1^b^	1.6E-156
Average branching interval	6.6 ± 1.0	3.4 ± 2.1	3.5 ± 1.4	7.1 ± 2.0	3.2 ± 1.6^b^	5.5E-76
No. of primary branches	7.4 ± 1.2	5.7 ± 1.1	5.3 ± 0.9	5.4 ± 1.4	5.2 ± 1.4	0.22
Yield per plant^a^	24.3 ± 4.1	14.0 ± 2.9	14.9 ± 3.1	18.5 ± 11.2	14.7 ± 8.4^b^	0.00011
1000-seed weight^a^	4.570 ± 0.304	3.426 ± 0.702	4.221 ± 0.394	4.364 ± 0.521	3.924 ± 0.660^b^	3.28E-13

^a^ Indicates that the trait with this sign was investigated in 20 individuals randomly sampled from dwarf and tall subpopulations, respectively

^b^ Indicates significant differences between dwarf and tall plants at the 0.01 level by *t*-test. Data are presented as mean ± standard deviation (SD)

### Construction of a high density SNP map

With the BC_1_ population derived from ‘ZS11’ × (‘ZS11’ × *Bndwf/dcl1*), we genotyped 109 plants. Although the Brassica 60 K SNP BeadChip has 52,157 SNP markers, after deleting invalid markers, only 14,682 polymorphic markers were used to construct the linkage map. Nineteen LGs obtained by JoinMap 4 software contained 818 bins representing 7489 markers (for detailed data see Additional file 2). The total length of the map was 1583.05 cM, the longest LG was C03 at 134.54 cM and the shortest LG was C09 at only 22.91 cM (Table 3). The mean interval between adjacent markers was 1.98 cM. This high-density map may be used for mapping the *BnDWF/DCL1* locus and the loci of other agronomic traits.

**Table 3:** Statistics of the LGs constructed from the BC_1_ population of *Brassica napus*

LG	Bin	Marker	Length (cM)	Mean interval (cM)	Max interval (cM)	Min interval (cM)
A01	51	403	75.67	1.51	9.67	0.11
A02	34	298	110.88	3.36	18.44	0.66
A03	66	439	133.96	2.06	16.06	0.27
A04	46	591	92.24	2.05	19.74	0.17
A05	66	630	95.63	1.47	12.14	0.10
A06	62	342	122.12	2.00	14.69	0.19
A07	48	323	71.47	1.52	7.85	0.17
A08	34	416	41.08	1.24	4.34	0.08
A09	66	599	119.57	1.84	11.19	0.21
A10	24	129	47.24	2.05	12.79	0.24
C01	33	447	86.53	2.70	19.09	0.23
C02	60	622	71.36	1.21	10.68	0.14
C03	61	628	134.54	2.24	18.00	0.38
C04	33	489	58.66	1.83	6.85	0.20
C05	26	187	64.75	2.59	13.37	0.44
C06	27	289	57.19	2.20	10.25	0.26
C07	34	242	65.09	1.97	10.50	0.36
C08	36	303	112.16	3.20	15.38	0.24
C09	11	113	22.91	2.29	9.48	0.68
Total	818	7489	1583.05			

### Mapping of *BnDWF/DCL1*

**Fig. 2:**
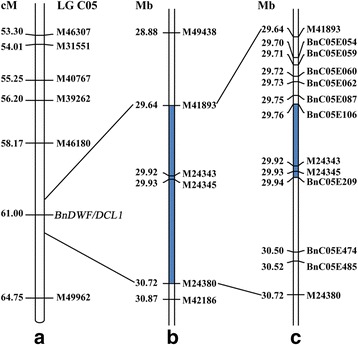
Mapping of *BnDWF/DCL1* with SNP and SSR markers using BC_1_ and F_5_ populations. **a** The *BnDWF/DCL1* locus was mapped by SNP marker at position of 61.00 cM of LG C05 in the BC_1_ population, which was in a 6.58-cM region between M46180 and M49962. **b** SNP screening of 29 F_5_ individuals narrowed the *BnDWF/DCL1* locus to the interval between M41893 and M24830 (1.08 Mb, the blue segment) of chromosome C05 in *B. napus* cv. ‘Darmor-*bzh*’, and this was co-segregated with M24343 and M24345. **c** SSR marker screening resulted in further mapping of the *BnDWF/DCL1* locus to the interval between SSR markers BnC05E106 and BnC05E209 (175 kb)

After building the saturated SNP genetic map, the *BnDWF/DCL1* locus was mapped onto LG C05, positioned in an interval of 6.58 cM between SNP bins M46180 and M49962 (Fig. 2a). A search of the *B. napus* genome database had shown that SNP probe sequences of the two flanking markers matched completely with their physical position on chromosome C05 in *B. napus* cv. ‘Darmor-*bzh*’*.* On the basis of the genome sequence of *B. napus* cv. ‘Darmor-*bzh*’, the *BnDWF/DCL1* locus is inferred to reside within a region of 16.354 Mb on C05 of *B. napus* cv. ‘Darmor-*bzh*’. LG C05 was 64.75 cM long and contained 26 bins representing 187 SNP markers, indicating that the chromosome C05 of *B. napu*s had enriched SNP markers in our population. Unfortunately, the target interval harbouring the *BnDWF/DCL1* locus did not have polymorphic SNP markers, leading to a failure in identifying the nearest recombination site by using this population.

### QTL mapping related to plant type

In addition to mapping of *BnDWF/DCL1*, we also mapped QTLs for agronomic traits in the BC_1_ population. As a result, we identified a single major QTL for plant height on LG C05, termed *qPHC05*, which explained 79.5 % of the phenotypic variation in plant height (Table 4). It apparently mapped at the same position as the *BnDWF/DCL1* locus, had a large LOD score of 35.0 (see Additional file 3), and had a high genetic effect (-62.30 cm). This probably indicates the *BnDWF/DCL1* locus (QTL) not only causes changes in leaf shape, but also reduces plant height in plants carrying the dominant *BnDWF/DCL1* locus. Furthermore, we identified a QTL for branching height (*qBHC05*), a QTL for main raceme length (*qMRLC05*), and a QTL for average branching interval (*qABIC05*). These QTLs of traits related to plant type were at approximately the same position as *BnDWF/DCL1*. They consistently had high LOD values (see Additional file 3), obvious genetic effects and high phenotypic variation explained (PVE) percentages (Table 4). Thus, we conclude that the *BnDWF/DCL1* locus (QTL) probably has a pleiotropic effect on plant type. In other words, the dominant locus *BnDWF/DCL1* causes elemental shrinkages in the plant architecture, leading to pleiotropic effects on different parts of plants. It is noted that the position of this dwarf locus together with its related traits are all different from the reported dwarf QTLs in *B. napus.*

**Table 4:** QTLs identified for agronomic traits in the BC_1_ population derived from parents ‘ZS11’ and *Bndwf/dcl1*

Trait	QTL	LG	Position	Marker interval	LOD	Effect	PVE (%)
Plant height	*qPHC05*	C05	61.1	M46180-M49962	35.0	−62.30	79.5
Branching height	*qBHC05*	C05	61.0	M46180-M49962	26.8	−20.28	71.2
Main raceme length	*qMRLC05*	C05	61.0	M46180-M49962	22.3	−27.37	65.2
Average branching interval	*qABIC05*	C05	61.5	M46180-M49962	10.9	−3.82	41.4

### Further mapping of the *BnDWF/DCL1* locus

The above-mentioned mapping interval for *BnDWF/DCL1* was too long to be of value for further studies due to the lack of polymorphic SNP markers in the mapping interval. To solve this problem, we investigated 29 F_5_ individuals derived from the cross between the European rapeseed cultivar ‘Tapidor’ and *Bndwf/dcl1.* Genotyping the 29 F_5_ individuals using the Brassica 60 K SNP BeadChip Array revealed that the *BnDWF/DCL1* locus co-segregated with SNP markers M24343 and M24345, and seven recombinants were found among the 29 F_5_ individuals. Of these, six recombinants with down-curved leaves were observed be recombined between M24343 and M41893, and one line with down-curved leaves was observed to show recombination between M24345 and M24380. Information from markers M41893, M24343, M24345 and M24380 helped narrow the mapping interval to a 1.08-Mb region between M41893 and M24380, and there were no other polymorphic SNP markers in this region (Fig. 2b).

On the basis of this revised mapping interval and its corresponding genome sequence, 155 simple sequence repeat (SSR) markers were designed to clarify the recombination site by polymerase chain reaction (PCR). Nine of the designed SSR markers were found to be polymorphic: BnC05E054, BnC05E059, BnC05E060, BnC05E062, BnC05E087, BnC05E106, BnC05E209, BnC05E474 and BnC05E485 (see Additional file 4). By genotyping the seven F_5_ recombinant individuals using these nine polymorphic markers, fortunately, we found that the *BnDWF/DCL1* locus can be mapped to the interval between BnC05E106 and BnC05E209, based on recombination site analysis with SSR markers. The relative orders and positions of all the polymorphic SNP and SSR markers on LG C05 and chromosome C05 of *B. napus* cv ‘Darmor-*bzh*’ are unanimous (Fig. 2). Accordingly, the mapping interval between BnC05E106 and BnC05E209 was identified to be 175 kb in length on basis of their positions on the C05 chromosome of *B. napu*s cv. ‘Darmor-*bzh*’ (Fig. 2c). No polymorphic SSR marker other than the nine SSR markers can be found in this small region, which was progressively determined by our careful investigation.

To validate these results, the remaining plants of the F_5_ family populations were genotyped using the SSR markers. PCR detection in the segregating or homologous F_5_ family populations produced results that were consistent with those of previous experiments, including phenotype observations and marker genotyping. Thus we may conclude that the *BnDWF/DCL1* locus is located in a 175-kb interval between BnC05E106 and BnC05E209 on chromosome C05 of *B. napu*s cv. ‘Darmor-*bzh*’.

Additionally, our analysis for the fine mapping of the *BnDWF/DCL1* locus was based on a formally released genome sequence database of *B. napus* cv. ‘Darmor-*bzh*’. Nevertheless, segments homologous to that harbouring the *BnDWF/DCL1* locus cannot be found in the genome database of *B. napus* cv. ‘ZS11’. However, in this database, we did find two highly homologous matches to the mapping segment, which were a segment of 86.78 kb in length on chromosome C04 (from 25534021 to 25620800), and a segment of 91.6 kb in length on chromosome C05 (from 29448801 to 29540400) (Additional file 5). Theoretically, it is of very small probability to depart two segments homologous to an entire segment by an evolutionary event at recent time. Thus, it may be inferred that the disparity between two public sources of genome information regarding this region is due to an error that occurred in assembly of the genomes of the allotetraploid species *B. napus*. However, strong systematic evidence from the aforementioned SNP and SSR marker experiments, such as recombination percentage and marker cosegregation information, consistently support our contention that the results based on the genome database of *B. napus* cv. ‘Darmor-*bzh*’ are reliable for this work. Analysis of the annotation information of the 175-kb mapping interval revealed that the chromosomal segments harbouring the *BnDWF/DCL1* locus in *B. napus* cv. ‘Darmor-*bzh*’ contain 16 annotated genes found in public databases (http://brassicadb.org/brad/downloadOverview.php) (Additional file 6).

## Discussion

Although studies of plant type regulation in plant species have been intensified, the new applicable gene is rare. Mining the key genes related to plant type is still of significance in many crops. Compact plant type may be used to increase planting density in *B. napus*, with the aim of elevating yield per unit area. A compact plant type together with the down-curved leaves phenotype was observed in a population derived from the *Bndwf/dcl1* mutant. The *BnDWF/DCL1* locus position and its related traits are all different from that of the reported dwarf QTLs in *B. napus* [16–25]. The *BnDWF/DCL1* locus has a more obvious effect on reducing plant height than these reported rapeseed dwarf loci and confers plants with compact architecture, thus, this will certainly be of value in breeding a variety with compact plant type because the moderate penalty of the dwarfism may possibly be compensated or overcome by increased planting density. However, in the future, the breeding potential of the *Bndwf/dcl1* mutant will need to be explored with attention paid to breeding strategies, although we still consider that it will be of significance in oilseed rape breeding.

Use of SNP markers has been beneficial to plant genotyping efforts because of the numerous distinct markers and high genome coverage [26–28]. In *B. napus*, the Brassica 60 K SNP BeadChip Array has recently helped advance rapeseed research efforts, and enabled the efficient construction of several high-quality saturated linkage maps over a short period [25, 29–32]. By using this SNP chip in the present study, we constructed a saturated *B. napus* map, with 818 bins containing 7489 markers and a total length of 1583.05 cM. The *BnDWF/DCL1* locus was primarily mapped to a 6.58-cM interval between M46180 and M49962 of *B. napus* chromosome C05. The SNP marker distance of 6.58 cM corresponds to the physical map length (16.354-Mb). This indicated that the mapping interval may contain a centromere since the physical distance is not consistent with the observed phenomena that 1 cM is on average equivalent to a genomic sequence length of 0.4–0.5 Mb in *B. napus*.

Although our investigations were initially limited by the lack of polymorphic molecular markers in the target mapping interval, we fortunately had alternative accessions that were derived from the cross between ‘Tapidor’ and *Bndwf/dcl1*, which have undergone recombination in the selfing and breeding process. The enriched materials prepared in this research helped us to progressively approach the target genes.

Compared with many other traditional mapping techniques, our mapping procedure included the additional step of homologous segment analysis. Indeed, this analysis is probably a necessary step in the mapping. Newly constructed public Brassica genome databases cannot entirely exclude the possibly of certain genome assembly errors, which may lead to the inaccurate mapping intervals. Analysis integrated with marker experimental information may help to perfect the mapping results, as this will clarify which sequences are more reliable in cases where there is disparity between two or more homologous genome sequence segments.

Various mechanisms underlying plant dwarfism have been reported. Genes participating in biosynthesis and signal transduction of plant hormones, such as GA, BR and auxin, have been related to dwarf plant architecture. Dwarfism may also be affected by homeotic genes and genes related to the cell wall, polyamine biosynthesis and transcription factors. The present study has revealed the genes within a designated mapping interval, which contains genes with functions similar to those reported to be associated with plant dwarfism. However, further studies are required to identify the gene responsible for the dwarf architecture with down-curved leaf mutant trait in *B. napus*.

## Conclusions

The *BnDWF/DCL1* locus was demonstrated to be associated with the dwarf architecture with down-curved leaf trait in *B. napus*. Construction of a high-density SNP map enabled us to position the *BnDWF/DCL1* locus in a 6.58-cM interval on LG C05 of *B. napus.* Further mapping with other materials derived from *Bndwf/dcl1* enabled us to narrow the interval for *BnDWF/DCL1* to 175-kb*.* QTL mapping indicated that *BnDWF/DCL1* to be a major dominant locus that has pleiotropic effects on leaf type and changes in plant type traits. Our findings have revealed a key locus with plant type breeding potential, and may offer insights into elucidating the molecular mechanism underlying the dominant plant type in *B. napus*.

## Methods

### Plant materials

The *B. napus* dwarf architecture with down-curved leaf mutant, *Bndwf/dcl1*, was originally isolated from an EMS-mutagenized *B. napus* pure line, NJ7982, at Nanjing Agricultural University, China. The dwarf architecture with down-curved leaf mutant was crossed with canola variety ‘ZS11’, and then backcrossed with ‘ZS11’ to produce the BC_1_ generation. We phenotyped the BC_1_ population plants at the seedling and mature stages. Fourteen normal plant DNA samples, 95 down-curved leaf plant DNA samples, and DNA from the recessive recurrent parent, ‘ZS11’, were then used for SNP genotyping. Two reciprocal F_2_ populations derived from the same cross were also used for genetic analysis.

One homologous selfing F_5_ family (contains 153 dwarf plants with down-curved leaves) and three segregating selfing F_5_ families (contain 14, 12 and 13 dwarf plants with down-curved leaves and 14, 8 and 5 normal plants with normal leaves, respectively) derived from the cross of “Tapidor × *Bndwf/dcl1*” were used to further map the dwarf architecture with down-curved leaf locus. Some of plants with dwarf architecture in the segregating families died because they were shadowed by taller plants, thus the data of the segregating families were not used for chi-square test. Twenty-nine individuals (23 dwarf plants with down-curved leaves and 6 normal plants with normal leaves) from the four F_5_ families were used for SNP marker genotyping with aim of further mapping the *BnDWF/DCL1* locus. All plants of these populations were used to identify recombination sites resulting from the breeding process, using SSR markers.

All materials were grown at the same density in fields of the Jiangpu Experimental Station at the Nanjing Agricultural University. Plants were sown uniformly in rows of 2.5 m length with 15 individuals in each row and 0.4 m spacing between rows. The BC_1_ and two reciprocal F_2_ populations were grown in 2012. The F_5_ lines were grown in 2014.

### Agronomic traits observation

In the BC_1_ population, agronomic traits, including plant height, branching height, main raceme length, number of first branches and average branching interval, were investigated in all the individuals, and 20 individuals randomly sampled from dwarf and tall subpopulations, respectively, were used to investigate the yield per plant and 1000-seed weight. Every eight individuals were randomly selected from the parents and F_1_ of the BC_1_ population to examine all the agronomic traits.

The plant height (*PH*) was measured from the ground to the top of the individual, the branching height (*BH*) was measured from the ground to the first node, and the main raceme length (*MRL*) was measured from the last branch base to the top of the plant. The average branching interval (*ABI*) was calculated using the following formula:

$$ ABI=\left(PH-BH-MRL\right)/\left( number\; of\; first\; branches-1\right). $$

### Construction of a SNP genetic map

Total DNA was extracted from fresh leaves using a modified cetyl trimethylammonium bromide (CTAB) method [33]. The DNA samples were diluted to 200 ng uL^−1^ and then genotyped using the Brassica 60 K SNP BeadChip Array. There are a total of 52,157 SNP markers in the Brassica 60 K SNP BeadChip Array, which is sufficient to genotype our DNA samples. DNA sample preparation, hybridization to the BeadChip and imaging of the arrays were performed by the Beijing Emei Tongde Development Co. Ltd (Beijing, China). Allele calling for each locus was performed using GenomeStudio genotyping software v2011.1 (Illumina, Inc.). Cluster definitions were based on genotype data from rapeseed individuals. The SNP markers were named using M plus index numbers assigned by GenomeStudio, which are presented in the text and their original names are listed in Additional file 2.

The polymorphic SNP markers were first sorted into different bins. The first marker within each bin was selected as the representative of the bin and was used to construct the linkage map with JoinMap 4 software [34]. The SNP markers were first grouped to different LGs at a recombination frequency of 0.22 using the “Population” function. The marker order and distances in each LG were calculated using the Regression mapping algorithm. The Kosambi function was used for calculating the cM map distances with a logarithm of odds (LOD) threshold of 1.0 and recombination frequency of 0.4.

### Mapping of the *BnDWF/DCL1* locus and QTLs for agronomic traits

A genetic map was constructed from the backcross population using the Brassica 60 K SNP BeadChip Array data. Detection of the *BnDWF/DCL1* locus and QTLs for plant type-related agronomic traits was performed for the BC_1_ population using the inclusive composite interval mapping (ICIM) method [35]. The LOD threshold for QTL detection was determined by permutation test analyses (1000 permutations, 5 % overall error level).

### Further mapping of the *BnDWF/DCL1* locus

On the basis of the constructed SNP map, the physical regions containing the *BnDWF/DCL1* locus were identified by aligning SNP probe sequences with the genomes of *B. napus* cv. ‘Darmor-*bzh*’ using BLASTN (http://blast.ncbi.nlm.nih.gov/). The locus of the *B. napus* cv. ‘Darmor-*bzh*’ genome, where the genome sequence has 100 % identity with the SNP probe sequence, is the perfect position of the corresponding SNP marker on it. The Brassica 60 K SNP BeadChip Array was used to genotype the 29 F_5_ individuals derived from the cross between ‘Tapidor’ and *Bndwf/dcl1*.

Following gene mapping by these methods, the interval sequences of approximately 1 Mb covering the significant SNP markers related to the mutant locus were downloaded from http://www.genoscope.cns.fr/brassicanapus/data/ for bioinformatics analysis. On the basis of the sequences of *B. napus* cv. ‘Darmor-*bzh*’ physical region containing the *BnDWF/DCL1* locus, 637 SSR loci were identified using SSRHunter 1.3 software [36] with a 6-bp motif maximum and three-repeat minimum. Of these, 155 SSR loci with a 150-bp sequence on both sides were selected to design primers using the Primer Premier 5.0 software [37] (see Additional file 7). The PCR conditions were as follows: denaturation at 95 °C for 10 min, followed by 35 cycles of 95 °C for 30 s, annealing for 40 s (the annealing temperature of each SSR marker is listed in Additional file 7), and 72 °C for 40 s, and a final extension step at 72 °C for 10 min.

The nine polymorphic markers identified from these 155 designed SSR markers were used for genotyping the F_5_ lines derived from a crosses between ‘Tapidor’ and the *Bndwf/dcl1* mutant, to determine the recombination site in the breeding materials with the aim of narrowing the mapping interval.

### Analysis of genes in the mapping interval of the *BnDWF/DCL1* locus

Whole genome sequences of *B. napus* cv. ‘Darmor-*bzh*’ were downloaded from public databases (http://www.genoscope.cns.fr/brassicanapus/) [38]. On the basis of the positions of SSR and SNP markers on the genome, the corresponding physical region of mapping interval could be obtained. A bioinformatics analysis of annotated genes in the mapping region was then completed.

## Additional files

Additional file 1: Table S1. Full list of the values of agronomic traits in the BC_1_ population. (XLSX 58 kb) Additional file 2: Table S2. Detailed SNP genetic map. (XLSX 296 kb) Additional file 3: Figure S1. Major QTLs for plant-type related traits mapped on LG C05 of the *Brassica napus*. (DOCX 89 kb) Additional file 4: Figure S2. Polymorphism identification of the SSR markers. (DOCX 1028 kb) Additional file 5: Figure S3. Dot matrix of the *BnDWF/DCL1* mapping interval of *B. napus* cv. ‘Darmor-*bzh*’ to *B. napus* cv. ‘ZS11’. (DOCX 284 kb) Additional file 6: Table S3. Genes on the mapped segments of chromosome C05 of *Brassica napus. (XLSX 22 kb)*
Additional file 7: Table S4. Information of designed SSR markers. (XLSX 25 kb)

## Abbreviations

‘ZS11’: ‘Zhongshuang11’
*Bndwf/dcl1*: Dwarf architecture with down-curved leaf mutant
BR: Brassinosteroid
CTAB: Cetyl trimethylammonium bromide
EMS: Ethyl methanesulfonate
GA: Gibberellic acid
ICIM: Inclusive composite interval mapping
LG: Linkage groups
LOD: Logarithm of odds
PCR: Polymerase chain reaction
QTL: Quantitative trait locus
SNP: Single-nucleotide polymorphism
SSR: Simple sequence repeat
