# Supplementary material for: Mapping a major QTL responsible for dwarf architecture in Brassica napus using a single-nucleotide polymorphism marker approach
Source: BMC Plant Biol. 2016 Aug 18;16:178. doi: 10.1186/s12870-016-0865-6 (PMC4991092; doi:10.1186/s12870-016-0865-6)
Supplement: Additional file 3: Figure S1. — Major QTLs for plant-type related traits mapped on LG C05 of the Brassica napus. (DOCX 89 kb) [file 12870_2016_865_MOESM3_ESM.docx]

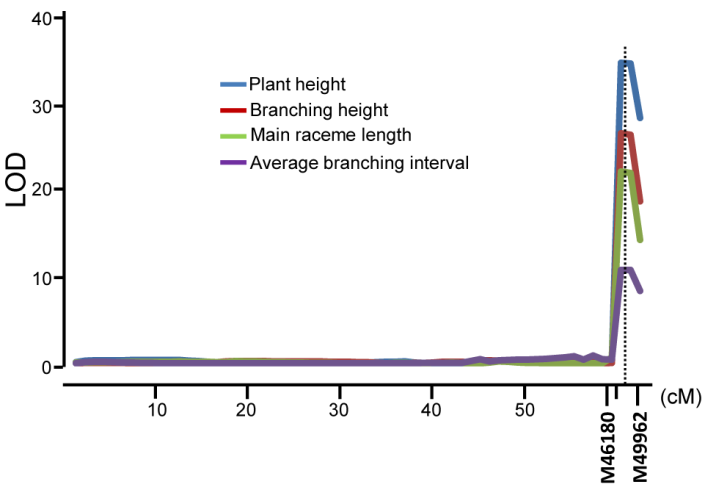


**Figure S1. Major QTLs for plant-type related traits mapped on LG C05 of the *Brassica napus*.** The LG C05 was 64.75 cM at length distributed in the horizontal ordinate. The QTLs for plant height, branching height, main raceme length, and average branching interval were all detected between M46180 and M49962.
