# Supplementary material for: Mapping a major QTL responsible for dwarf architecture in Brassica napus using a single-nucleotide polymorphism marker approach
Source: BMC Plant Biol. 2016 Aug 18;16:178. doi: 10.1186/s12870-016-0865-6 (PMC4991092; doi:10.1186/s12870-016-0865-6)
Supplement: Additional file 4: Figure S2. — Polymorphism identification of the SSR markers. (DOCX 1028 kb) [file 12870_2016_865_MOESM4_ESM.docx]

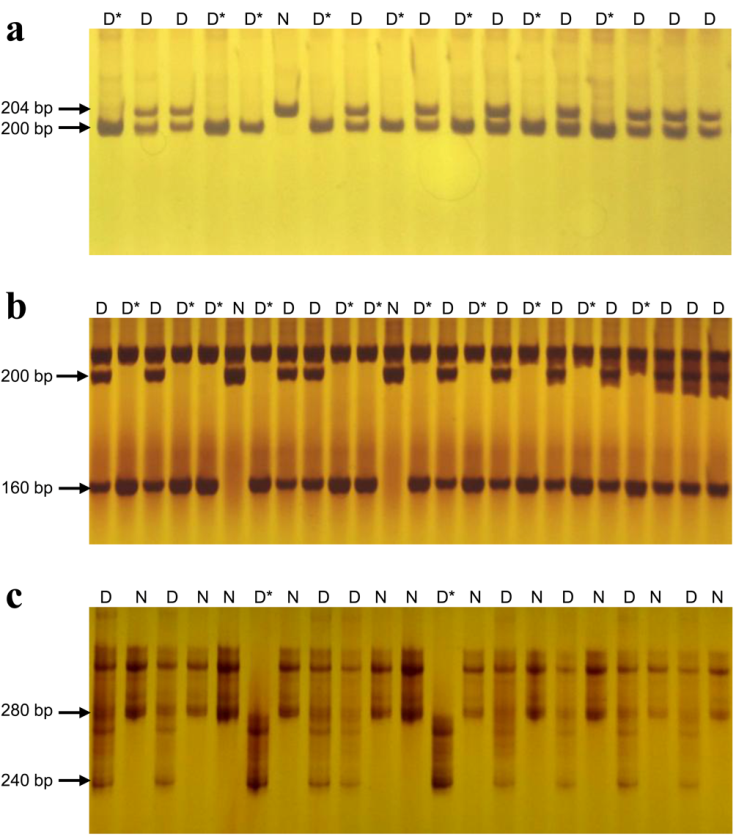


**Figure S2. Polymorphism identification of the SSR markers.** PCR products of SSR co-dominant markers BnC05E059 (**a**), BnC05E106 (**b**) and BnC05E209 (**c**), in the germplasm population, ‘D*’, ‘D’ and ‘N’ indicate product from homozygous down-curved leaf plants, heterozygous down-curved leaf plants and homozygous normal plants in the germplasm population, respectively.
