# Supplementary material for: Mapping a major QTL responsible for dwarf architecture in Brassica napus using a single-nucleotide polymorphism marker approach
Source: BMC Plant Biol. 2016 Aug 18;16:178. doi: 10.1186/s12870-016-0865-6 (PMC4991092; doi:10.1186/s12870-016-0865-6)
Supplement: Additional file 5: Figure S3. — Dot matrix of the BnDWF/DCL1 mapping interval of B. napus cv. ‘Darmor-bzh’ to B. napus cv. ‘ZS11’. (DOCX 284 kb) [file 12870_2016_865_MOESM5_ESM.docx]

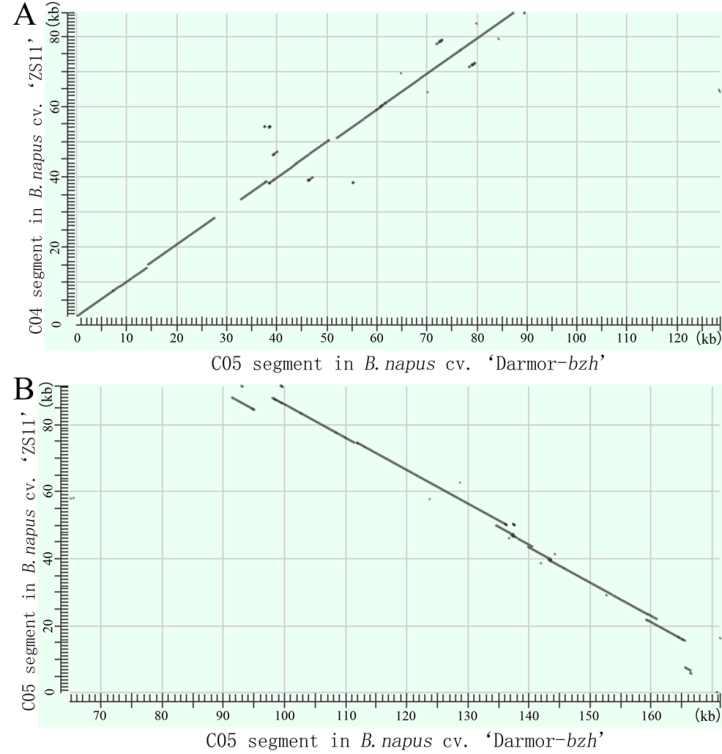


**Figure S3. Dot matrix of the *BnDWF/DCL1* mapping interval of *B. napus* cv. ‘Darmor-*bzh*’ to *B. napus* cv. ‘ZS11’. A.** The 86.78-kb C04 segment in *B. napus* cv. ‘ZS11’ (y-axis) has similarity to 0-90 kb of the the *BnDWF/DCL1* mapping interval of chromosome C05 of *B. napus* cv. ‘Darmor-*bzh*’ (x-axis). **B.** The 91.6-kb C05 segment in *B. napus* cv. ‘ZS11’ (y-axis) has similarity to 90-172 kb of the the *BnDWF/DCL1* mapping interval of chromosome C05 of *B. napus* cv. ‘Darmor-*bzh*’ (x-axis).
